# Exploring Angiosperms353: Developing and applying a universal toolkit for flowering plant phylogenomics

**DOI:** 10.1002/aps3.11443

**Published:** 2021-07-26

**Authors:** Angela J. McDonnell, William J. Baker, Steven Dodsworth, Félix Forest, Sean W. Graham, Matthew G. Johnson, Lisa Pokorny, Jennifer Tate, Susann Wicke, Norman J. Wickett

**Affiliations:** ^1^ Negaunee Institute for Plant Conservation Science and Action Chicago Botanic Garden 1000 Lake Cook Road Glencoe Illinois 60022 USA; ^2^ Royal Botanic Gardens, Kew Richmond Surrey TW9 3AE United Kingdom; ^3^ School of Life Sciences University of Bedfordshire University Square Luton LU1 3JU United Kingdom; ^4^ Department of Botany University of British Columbia 6270 University Boulevard Vancouver British Columbia V6T 1Z4 Canada; ^5^ Department of Biological Sciences Texas Tech University Lubbock Texas 79409 USA; ^6^ Centre for Plant Biotechnology and Genomics (CBGP) UPM‐INIA‐CSIC 28223 Pozuelo de Alarcón (Madrid) Spain; ^7^ School of Fundamental Sciences Massey University Palmerston North 4442 New Zealand; ^8^ Plant Evolutionary Biology Institute for Evolution and Biodiversity University of Münster Münster Germany; ^9^ Plant Systematics and Biodiversity Institute for Biology Humboldt‐Universität zu Berlin Berlin Germany

Target enrichment represents a useful, cost‐effective method for researchers working on the phylogenomics of non‐model organisms (e.g., Cronn et al., 2012; Hale et al., 2020). The ability to sequence a customizable predefined genomic subset for several dozens or even hundreds of taxa allows in‐depth analyses and the testing of phylogenetic hypotheses in ways that were not previously possible (reviewed in McKain et al., 2018). The most popular methods for targeted sequencing of genomic loci in phylogenomics include (long‐)amplicon sequencing (Rothfels et al., 2017) and hybridization capture (Mandel et al., 2014; Weitemier et al., 2014). Targeted amplicon sequencing is based on single‐fragment PCR amplification or by using multiplexing methods such as a microfluidic PCR‐based amplification of multiple pre‐selected genomic regions (e.g., Zhang and Ozdemir, 2009; Ho et al., 2014), which can then be pooled and sequenced. Massively parallel amplicon sequencing was first used in medical diagnostics (Turner et al., 2009) and was later applied to metazoan phylogenetics (Bybee et al., 2011; O’Neill et al., 2013). Microfluidic PCR and long‐amplicon sequencing were subsequently applied in plant systematics (Uribe‐Convers et al., 2014, 2016; Gostel et al., 2015). Amplicon‐based methods can be time consuming as they require careful optimization and validation of primers. These methods are also susceptible to many of the common problems in PCR (such as nonspecific products, inability to amplify large loci in their entirety, or simply no products). Recently, amplicon approaches have been largely supplanted by hybridization‐based targeted enrichment, which allows for relatively rapid probe design with reference to a few related transcriptomes or genomes, and allows simultaneous and efficient recovery of many hundreds of genes.

Target enrichment via hybridization‐based sequence capture can use customized or universal probes (short nucleotide fragments between 80 and 120 bp in length, also called baits). This versatile and powerful approach relies on probes to capture complementary sets of genomic targets from whole genome DNA or cDNA samples using solid‐phase or in‐solution hybridization (Gnirke et al., 2009; Mamanova et al., 2010; Cronn et al., 2012). To account for frequent (ancient) whole genome duplication events, plant systematists can identify sets of consistently low‐to‐single‐copy markers that balance two common issues: ensuring recovery across a broad range of taxa, while containing sufficient phylogenetic informativeness. Target regions can be customized to address specific research questions, including targeting genes common to a metabolic pathway (Gardner et al., 2016; Folk et al., 2021) or genes of agronomic interest (Witek et al., 2016; Soto Gomez et al., 2019). The targets are often complete genes or exonic sequences, but non‐coding DNA such as introns or untranslated regions can also be enriched. In‐solution capture of the probe targets depends on the thermodynamics of hybridization. This process is governed, in part, by the DNA complexity of the bait and the probe length. Longer probes tolerate more mismatches, making them more suitable for capture across different species. Higher tiling density (the degree to which probes overlap, e.g., 75%) can compensate for failures of specific probes to hybridize or for low‐quality input material, such as those stemming from historical herbarium specimens, so‐called herbariomic approaches (Brewer et al., 2019). While overlapping probes increase the chance of capturing the target of interest via neighboring probes, there is a trade‐off, as a higher tiling density tends to reduce the number of loci that can be examined per capture kit.

Targeted enrichment of nuclear genes also overcomes several potential drawbacks of estimating phylogenies from organelle data alone. The single‐locus behavior of organellar DNAs means that only a single history is provided: the entire plastid genome is expected to function as a single linkage group (Birky, 2001; but see Zhang et al., 2020). Organellar data are also prone to high rates of genetic drift in small population sizes, which could lead to a less accurate estimate of lineage differentiation, compared with large sets of nuclear genes (reviewed in Wicke and Schneeweiss, 2015). Although target capture data provide many more gene histories, analysis of these data requires specific bioinformatic approaches, including assembly of gene sequences without genomic references and detection of local gene duplication events that violate assumptions of orthology. In this regard, the plant systematics community has been innovative in addressing the related computational challenges, resulting in the wide adoption of targeted sequencing for plant systematics in recent years.

Angiosperms353 is a tool for enabling phylogenomic‐level analysis of any angiosperm group at any scale; the targeted genes and associated probes (Johnson et al., 2019) were developed from a set of low‐copy nuclear genes used to infer the phylogeny of all green plants (One Thousand Plant Transcriptomes Initiative et al., 2019) to facilitate the collection of phylogenomic data in a relatively cost‐effective and repeatable way. When considering Angiosperms353, it is important to distinguish between the low‐copy orthologous genes found in all angiosperms and the 120‐bp probe sequences used to enrich genomic libraries. The genes were selected based on copy number (ideally, genes that are single copy across more than 1400 available plant transcriptomes) and contain useful phylogenetic signal at deep and recent time scales. The probe sequences were designed from ~600 angiosperm transcriptomes and genomes, and were selected to maximize sequence similarity to other angiosperms, while minimizing the number of sequences used for probe design. Distinguishing the genes from the probes is important when considering the advantages, limitations, and future directions for Angiosperms353 in phylogenetic systematics, as discussed below.

Although initial reports of the utility of Angiosperms353 genes and probes have been promising (Dodsworth et al., 2019), a few key questions remain: (1) Can Angiosperms353 probes truly be used to reliably recover orthologous genes across all groups of flowering plants?; (2) Is an angiosperm‐wide probe design the most efficient for all targeted sequencing projects?; (3) What new bioinformatics tools are needed to apply Angiosperms353 genes to previously intractable research questions?; and (4) Are Angiosperms353 genes variable enough within species to extend the utility of the probes to population genetic studies? In this special issue and its companion issue in the *American Journal of Botany* (Baker et al., 2021b), the papers collected address these questions and demonstrate a number of ways in which research employing Angiosperms353 will benefit future angiosperm phylogenomic studies. This issue of *Applications in Plant Sciences* is focused on a variety of methodological issues, including comparisons between the Angiosperms353 probe set and taxon‐specific probe sets, the development of new tools for specific lineages, a new lab technique to enrich libraries using Angiosperms353 and taxon‐specific probe sets simultaneously, and bioinformatic approaches to facilitate careful assembly and analysis of data obtained using the Angiosperms353 kit.

## PROBE SET COMPARISONS AND DEVELOPMENT OF NEW TOOLS

Low‐to‐single‐copy nuclear markers have been an important tool in evolutionary studies of angiosperms (e.g., Small et al., 2004; Wu et al., 2006; Duarte et al., 2010). The identification of a set of hundreds of nuclear markers shared across angiosperms has facilitated the design of probes for targeted enrichment and subsequent high‐throughput sequencing (Johnson et al., 2019). In this issue, three papers address the application and expansion of the Angiosperms353 loci. These papers build on existing resources to design new lineage‐specific probes that contain the Angiosperms353 loci (Eserman et al., 2021; Ufimov et al., 2021), generate careful comparisons of the performance of an existing lineage‐specific probe set with the Angiosperms353 probe set (Siniscalchi et al., 2021), and investigate the efficacy of simultaneous enrichment of libraries using universal and lineage‐specific probes in a single hybridization reaction (Hendriks et al., 2021).

Eserman et al. (2021) describe the development of Orchidaceae963, a new bait set that incorporates 254 of the Angiosperms353 loci, which they tested on members of three subfamilies of Orchidaceae. This resource will facilitate systematic work in the family and is also expected to be useful in population and conservation genomic studies. The incorporation of the Angiosperms353 loci along with custom marker genes is becoming more common (Yardeni et al., 2019; Jantzen et al., 2020; Christe et al., 2021; Ogutcen et al., 2021), and will allow collaboration between research teams and foster data sharing across studies. Comparative analyses of the custom sets and the Angiosperms353 set will naturally follow (e.g., see Larridon et al., 2020; Shah et al., 2021). For example, Ufimov et al. (2021) found a similar amount of parsimony informative sites using the Angiosperms353 probe set and a custom probe set in their study of Rosaceae subtribe Malinae. They also showed that the custom probes allowed for improved locus recovery rates across samples and lower levels of gene tree discordance (but see Yardeni et al., 2019). Siniscalchi et al. (2021) compared their lineage‐specific Compositae1061 probes with the Angiosperms353 probes in eight members of the sunflower family. Although they were able to recover more loci on average with the lineage‐specific probes, they found lower levels of paralogy in the gene trees based on the Angiosperms353 loci, which may simplify downstream analyses of the latter versus the former. Hendriks et al. (2021) performed a test case for combining both lineage‐specific (i.e., Brassicaceae) and universal probes in a single hybridization reaction to simultaneously enrich libraries for both sets of loci. They found high levels of enrichment and locus recovery for both sets of targets across 26 Brassicaceae samples that included 16 samples from a single tribe and 10 samples reflecting broader sampling across the family. Their study shows that it is possible to generate data for multiple probe sets with little extra cost or work per sample.

Taken together, these papers highlight that researchers have several options when choosing loci for target capture in flowering plants. The Angiosperms353 genes provide a ready set of loci that are low copy and reliably recovered using the universal Angiosperms353 probes, or by using custom probes designed with orthologs of the Angiosperms353 genes from the target taxa. The feasibility of combining probe sets during hybridization suggests additional flexibility. There is a trade‐off, as custom probe designs will have higher per‐sample costs and require existing sequence data in the focal group. However, far fewer probes will be needed for the Angiosperms353 genes in a custom design, allowing for additional loci for a given number of probes. Whichever choice is made, by including Angiosperms353 in a target capture project, researchers will benefit the entire phylogenetics community by enabling meta‐analysis that parallels the broad adoption of single‐gene markers in past decades.

## NEW INNOVATIONS: BIOINFORMATICS

The development of Angiosperms353 into a common tool for molecular phylogenetics and population genetics has been paralleled by the need for new analytical workflows. One of the most active areas of tool development is in using extensions to existing tools to facilitate analysis of Angiosperms353 data. In this issue, three tools specifically tie in with HybPiper (Johnson et al., 2016), a popular tool for assembly and recovery of genes and flanking regions from target‐capture sequencing reads. A common issue identified with Angiosperms353 data assembled using HybPiper is relatively low sequence recovery (e.g., Gaynor et al., 2020), but it is often unclear whether the issue lies with the molecular tools (i.e., poor hybridization between probes and target DNA) or bioinformatic tools (i.e., poor recovery in HybPiper or other tools). To test this, McLay et al. (2021) developed new target files to improve the mapping frequency of reads to Angiosperms353 target genes, a result independently confirmed by Slimp et al. (2021) and Lee et al. (2021). McLay et al. (2021) also provide a workflow that uses a hidden Markov model to choose clade‐specific target sequences from a database of potential Angiosperms353 sequences. Their results indicate that the sequence divergence between the Angiosperms353 probe sequences and the target DNA of angiosperms during nucleic acid hybridization and subsequent sequencing is more forgiving than the effect of the same divergence on gene recovery during bioinformatic analysis. For researchers struggling with poor target enrichment efficiency using Angiosperms353, McLay et al. provide a potential bioinformatic solution that may reduce the need to re‐enrich libraries. Their approach is to improve the target file used to recover genes in HybPiper by selecting more appropriate orthologs of the Angiosperms353 genes.

Nauheimer et al. (2021) describe HybPhaser, a method for detecting hybrid taxa using HybPiper assemblies. Using an Angiosperms353 data set collected for *Nepenthes*, they employ an innovative two‐step strategy in which an initial phylogeny is first used to identify representatives of different clades that have low heterozygosity (and are thus presumably not hybrids). In the second step, reads from putative hybrids are mapped to these chosen clade representatives. Differential mapping information is then used to phase hybrid sequences, which can then be placed on a multiply‐labeled tree to identify hybrid origins. The HybPhaser method also has promise for addressing a common issue in target‐capture phylogenetics: the identification of paralogous genes. This builds upon previous work using clade references to identify paralogs (Gardner et al., 2021), but the development of a more generalizable workflow in HybPhaser will be of use to many researchers unsure about whether target‐captured genes are single copy in their data.

Slimp et al. (2021) also describe new methods for downstream analysis of HybPiper output, for use in population genomic studies and the calculation of within‐species demographic parameters. Their method adapts previously described workflows (Kates et al., 2018) to use HybPiper “supercontigs” (targeted coding regions and flanking non‐coding regions) as reference sequences within species. Within‐species variants can then be assessed jointly and used with standard population genomics programs (also see Beck et al., 2021 and Wenzell et al., 2021). Slimp et al. further demonstrate that the Angiosperms353 loci are variable within populations of several species, which suggests that genome‐scale estimation of population parameters is now feasible without the need to develop taxon‐specific methods.

## THE FUTURE OF TARGETED ENRICHMENT IN ANGIOSPERM PHYLOGENOMICS

Since its initial publication, there has been a rapid and substantial uptake of Angiosperms353 to address systematics questions. The papers in this issue and its companion *American Journal of Botany* issue further illustrate that the utility of data generated through target enrichment extends beyond phylogenomics, with promising applications into population and conservation genomics. Of course, with the adaptability of data comes a requirement for flexible and innovative methods to process and analyze these data. While new methods and novel applications are already emerging, we recognize that there are some challenges and improvements that lie ahead.

The need to evaluate the efficiency of recovering the targeted loci, both in vitro and in silico, is reflected in several papers in this issue, highlighting the distinction between targeted Angiosperms353 genes and the probes used to recover them. The papers presented here and in the companion issue build further evidence that the Angiosperms353 genes can be reliably recovered across the diversity of flowering plant species, with strong phylogenetic signal at scales ranging from deep time to the population level. Several papers in this issue describe innovations in how these target genes are recovered. Custom probe sets can still provide additional phylogenetic resolution within focal taxa by capturing more genes with the same number of probes, but the inclusion of Angiosperms353 loci along with custom probe sets will enable meta‐analyses to compare species across flowering plants. In silico, the use of enhanced target files offers potential improvements for the assembly of enriched sequence libraries and illustrates that newly available genomic data can be used to complement the original Angiosperms353 target file and probe set. In this regard, the availability of an ever‐increasing number of complete flowering plant genome and transcriptome sequences (e.g., One Thousand Plant Transcriptomes Initiative et al., 2019), and the massive increase in available sequence data facilitated by Angiosperms353, should allow for future refinement of the probes and choice of target sequences to better represent the diversity of angiosperms and maximize the length of sequences recovered across a broader set of taxa, including those with extremely altered lifestyles that affect the rate of sequence evolution, such as in heterotrophic taxa (e.g., Lam et al., 2018). Of course, the utility of Angiosperms353 data, or any other set of target enrichment data, relies on the accessibility of these genomic resources.

Currently, there is no central data repository specifically tailored to access target enrichment data in a way that is not taxon specific. The Kew Tree of Life Explorer (https://treeoflife.kew.org/) was recently launched (Baker et al., 2021a) to make freely available pre‐ and post‐publication Angiosperms353 data for thousands of taxa. The continued development of accessible tools to store and distribute these data will accelerate the progress that has been made thus far. For example, the ability to download a specific set of genes for a specific set of taxa from a central repository, rather than having to download and then filter multiple data sets from publication‐specific repositories, would significantly improve the ability to incorporate existing data for addressing new research questions. Similarly, a centralized repository for uploading assembled and annotated target enrichment data generated for any taxon across the entire tree of life facilitates standardization and accessibility. A streamlined set of tools for identifying Angiosperms353 homologs for building custom probe sets would also increase the efficiency of data collection, much like how MarkerMiner (Chamala et al., 2015) provides a way to identify orthologs from a starting data set. The adoption of Angiosperms353 and target enrichment more generally in angiosperm systematics should cultivate further growth in data and in methods, as we have seen in this special issue. As more people use these data and methods, the opportunities for positive developments will only increase further, and improvements to existing probe sets, the development of new probe sets, opportunities to create solutions to challenges such as the resolution of paralogs, and the establishment of companion databases are all likely to be addressed head‐on and embraced by the angiosperm systematics community.
